# Development and External Validation of a Machine Learning Model for Classification of Mild Cognitive Impairment and Dementia Using Clinical Data

**DOI:** 10.3390/medicina62071356

**Published:** 2026-07-14

**Authors:** Davis Kannenieks, Zanda Priede, Andrejs Millers, Karlis Kristofers Velins

**Affiliations:** 1 Faculty of Medicine, Riga Stradins University, LV-1007 Riga, Latvia; 2 Department of Neurology and Neurosurgery, Riga Stradins University, LV-1002 Riga, Latvia; zanda.priede@rsu.lv (Z.P.); andrejs.millers@rsu.lv (A.M.); 3 Department of Mathematics, Imperial College London, London SW7 2AZ, UK; kvelins@gmail.com

**Keywords:** subjective cognitive decline, mild cognitive impairment, early dementia detection, machine learning algorithm, NACC dataset, SHAP analysis, XGBoost classification

## Abstract

*Background and Objectives*: As society ages, the number of patients with cognitive impairment is increasing. Machine learning methods that use structured clinical and cognitive-assessment data during routine diagnostic work-up may support and monitor structured classifications of cognitive status. This kind of approach can improve early screening, reduce physicians’ workload and develop greater support for personalized treatment. To develop an XGBoost-based machine learning model using the National Alzheimer’s Coordinating Center (NACC) dataset and to evaluate the model’s precision with clinician-assigned diagnosis in a Latvian retrospective cohort study. *Materials and Methods*: The research was designed as a retrospective external validation cohort study that used two data sources. Firstly, the National Alzheimer’s Coordination Center (NACC) longitudinal dataset was used to train the ML model. Secondly, medical records gathered from Pauls Stradins Clinical University Hospital dating from 2020 to May 2025 were used to evaluate the algorithm’s precision. *Results*: In the NACC study, the weighted four-class model achieved an overall accuracy of 84.0% and a balanced accuracy of 70.9%, but the SCD class remained poorly classified. After reframing the model to a three-class model the performance grew stronger for normal cognition, mild cognitive impairment (MCI) and dementia. Class distribution in the Latvian cohort consisted of dementia (*n* = 138); MCI (*n* = 13); and subjective cognitive decline (SCD) (*n* = 2). Dementia was identified most strongly—124/138 (sensitivity—89.9%). MCI was correct in 9/13 cases (sensitivity—69.2%). SCD cases were excluded. Overall, the model agreed with the neurologist-assigned diagnoses in 88.1% of the cases (133/151). *Conclusions*: The ML classification model has high precision when comparing with neurologist-assigned diagnoses, but it struggles to separate adjacent early-stage diagnoses, meaning that it did not reliably identify SCD. These findings support further methodological development and the implementation of prospective research. Nevertheless, this technology has high potential for being integrated in the future to aid triage and early screening, especially when advanced diagnostics are limited.

## 1. Introduction

Cognitive function is a mental process that enables individuals to acquire, understand and utilize information through thinking, experience and sensory input [1]. It is necessary for a better quality of life, independence, and social participation. Although cognitive aging is a natural part of the human lifespan, cognitive-associated diagnoses are becoming increasingly important in both medicine and public health. According to the World Health Organization (WHO), the global population aged 60 years and older reached 1 billion in 2020 and is projected to increase to 2.1 billion by 2050 [2]. Consequently, age-associated neurodegenerative diseases are expected to rise substantially.

Alzheimer’s disease (AD) is the most common cause of dementia and is characterized by progressive, age-related cognitive decline that affects not only the patient but also the surrounding people, family, friends and caregivers. Importantly, the pathogenesis of AD begins many years, often decades, before the first clinical symptoms become apparent. In this preclinical period, biochemical and structural brain changes accumulate, which do not necessarily correlate with clinical symptoms at first. As a result, the diagnosis is frequently delayed until measurable objective data can be obtained for cognitive deficit, at which point treatment options are more limited [3,4].

The clinically relevant stage that may improve early detection, and therefore guide patients to faster lifestyle modifications and closer clinical monitoring, is the period before objectively measurable cognitive impairment, commonly referred to as subjective cognitive decline (SCD) [5]. SCD describes a condition in which individuals report worsening memory, orientation, or concentration, while objective cognitive testing does not yet demonstrate clear abnormalities [6,7]. Historically, such complaints were often considered part of normal aging. However, more recent evidence suggests that SCD may be associated with early manifestations of AD, particularly in individuals with a relevant family history or other risk factors [6].

At the same time, SCD assessment and diagnosis remains challenging in clinical settings, due to evaluation that relies on patient-reported symptoms and physician interpretations, both of which introduce high subjectivity and the possibility of errors [5]. Although a broad range of diagnostic tools are available, for instance, biomarkers and neuroimaging [8], they are not always accessible and may not be clinically or economically justified for an early-stage diagnostic evaluation.

Recent advances in clinical technologies and data analytics have created new possibilities to support physicians in their diagnosis, risk stratification and prognosis. Artificial intelligence (AI), more specifically machine learning (ML), is one of these technologies that can integrate and analyze structured/tubular data (cognitive test results, laboratory findings) and unstructured data (radiological images, medical history records, and clinical notes) to identify patterns that can reveal clinically meaningful associations that are difficult to detect through standard assessments [9,10]. This technology also comes with its limitations and challenges—trusting the algorithm outputs/results, the “black box” phenomenon, data interpretability and quality [11]. This produces a reasonable question: whether ML should be viewed as a replacement for or a supporter of physicians in clinical judgments and early cognitive assessments. Therefore, this research will externally validate a supervised ML model against clinician-assigned diagnoses that may help to identify the strengths and limitations of this technology and provide a clearer analysis of how effective it is.

### 1.1. Aim of the Research

The aim of this research is to develop an XGBoost-based machine learning model using the National Alzheimer’s Coordinating Center (NACC) dataset and to evaluate the model’s precision with clinician-assigned diagnosis in a Latvian retrospective cohort study, assessing whether the model can reliably distinguish between dementia, MCI, and SCD using structured clinical and cognitive-assessment variables during routine diagnostic work-up, without the use of biomarkers or neuroimaging.

### 1.2. Research Objectives

1. To train an ML classification algorithm to distinguish between SCD, MCI and dementia using a prospective international cohort dataset from the National Alzheimer’s Coordinating Center [12].
2. To externally evaluate the accuracy between an ML algorithm and neurologist-assigned diagnoses in patients with mild cognitive impairment and risk of Alzheimer’s disease.
3. To identify the clinical and cognitive parameters that strongly contribute to ML-based predictions using SHAP (Shapley Additive Explanations) analysis.
4. To evaluate the model’s performance when diagnostic-assessment-derived features have been removed.
5. To describe class-specific model performance across different cognitive status categories using confusion matrices and class-wise evaluation metrics.

## 2. Materials and Methods

### 2.1. Study Design

The study was designed as a retrospective external validation cohort study. The main purpose was to compare the diagnostic category produced by the machine learning model with neurologist-assigned diagnosis, and to evaluate the model’s agreement with the clinical diagnosis. This study should not be interpreted as a comparison between an ML model and a physician. The model was developed to explore whether using only subjective and objective clinical data, without biomarker testing or radiological imaging findings, could support the classification of cognitive-status categories.

Two datasets were used. A prospective international cohort longitudinal dataset from the National Alzheimer’s Coordinating Center (NACC, Seattle, WA, USA) was used to develop and train the ML model. The data are contributed by the NIA (National Institute of Aging, Bethesda, MD, USA)-funded Alzheimer’s Disease Research Centers (ADRCs) [12]. A retrospective Latvian clinical cohort from Pauls Stradins Clinical University Hospital (PSCUH, Riga, Latvia) was used for external evaluation against clinician-assigned diagnosis. The cohort design approach was selected because it allowed structured assessment of diagnostic categories, but it was limited by the severe class imbalance, due to the lack of cognitively diverse diagnoses (SCD and MCI) during the retrospective research period (from 2020 to May 2025).

### 2.2. Data Sources and Baseline Characteristics

For the model development, data were obtained from the NACC, which provides standardized longitudinal clinical information. The data includes repeated clinical visits; structured cognitive and functional assessments; and demographic, clinical and psychometric information seen in Table 1, supporting the model’s learning across diverse patient populations. Access to NACC data is restricted due to the NACC Data Use Agreement, and the dataset cannot be redistributed by the authors; qualified researchers may obtain access by submitting a data request to the NACC and completing the required agreements.

To evaluate the model’s accuracy, the Latvian retrospective dataset (see the baseline characteristics in Table 1) was obtained from PSCUH and covered January 2020 to May 2025. Patients were included if a diagnosis matched the cognitive status category (subjective cognitive decline; mild cognitive impairment; dementia) of this research and was diagnosed within this period. The last clinical visit at which the diagnosis was established and recorded was used to assess the model’s accuracy. Patients whose diagnostic endpoint fell outside the period were excluded from the research.

### 2.3. Ethical Approval

The research was approved by the Riga Stradins University Ethics Committee (No. 2-PĒK-4/952/2025) and authorized by the PSCUH Scientific Institute. Patient confidentiality and privacy were maintained according to institutional requirements, and analyses were performed using encrypted data.

### 2.4. Diagnostic Categories

The machine learning (ML) model was configured to output four diagnostic categories: normal cognition; subjective cognitive decline (SCD); mild cognitive impairment (MCI); and dementia. In the Latvian cohort study, reference labels were derived from the International Classification of Diseases (ICD) codes recorded in the clinical documentation. The model did not attempt to distinguish dementia types, so all dementia etiologies were grouped as “dementia”.

In the PSCUH dataset, the neurologist-assigned diagnoses were based on standard neurological assessment, available clinical documentation (patient and/or informant history, cognitive screening result when available, functional assessment, comorbidities named with ICD codes and medication history). In this cohort, MoCA (The Montreal Cognitive Assessment) and CDR (The Clinical Dementia Rating) tests were available for a subset of patients, while MMSE (The Mini-Mental State Examination) was not available for any of the patients. No separate study-specific diagnostic information, protocol or tools were applied retrospectively.

### 2.5. Data and Variables Used

The data and variables were selected to reflect clinically accessible information that would be meaningful for cognitive-status classification [5,13]. The full variables include sociodemographic information (sex, age, education, marital status, living status, economic situation); patient-/member of patient’s family-reported cognitive complaints; objective cognitive measures (MoCA, MMSE, CDR tests and CDR domains); psychiatric (depression and anxiety-related measures); vascular/somatic risk factors (hypertension, diabetes mellitus, stroke and heart attack history); comorbidities; and the use of medication (type of medication). No other standard protocols were used in this research. Because MoCA, MMSE, CDR and CDR domain variables are also commonly used by clinicians when assigning diagnostic categories, these variables may partly encode the diagnostic process itself. This potential circularity was considered in the preparation of the model and in SHAP analysis results. Therefore, a separate model was built to assess the impact of the MoCA, MMSE, CDR and CDR-domain scores.

In the NACC dataset, the final feature list used to train the model was determined by selecting only the predefined variables (mentioned above) that were also present in the Latvian cohort study, ensuring consistent data output without including additional variables that could influence the model’s accuracy.

### 2.6. Data Processing and Handling of Missing Values

Data preparation and modeling were implemented using a structured machine learning pipeline. The predictor matrix was constructed from the selected feature set, and the target variable was defined as “Diagnosis.” Medical records missing the target diagnosis label were removed before training.

In both datasets, many entries contained missing or unknown data. In the NACC dataset, originally missing or unknown values were sometimes encoded as random numbers. For example, for a binary column having values of 0 and 1, the value 9 was assigned in the case of ‘unknown’. Adjustments for all variables that contained such numbers were made as the model would otherwise treat it as a continuous value which would be 9 times larger than the answer ‘yes’ coded as 1 which would cause noise injected as a signal into the model and reduce performance. Adjustments were made on a case-by-case basis, either setting the missing values to NaN (Not a Number) for numerical features or distinguishing another categorical class such as ‘unknown’ for the categorical features [14].

To accommodate different data types, preprocessing was performed using a ColumnTransformer with separate pipelines for numerical and categorical features. The numerical variables were imputed using the median value and then standardized. Categorical variables were imputed using the most frequent value and transformed using one-hot encoding with handling of previously unseen categories [15].

In the NACC dataset the 4 classes were heavily imbalanced. While normal cognition made up 48.9% of all entries, SCD accounted for only 4.4% of the total dataset, MCI for 17.5% and dementia for 29.2%. To adjust for the imbalance and to reduce bias, multiple bias-reducing approaches such as SMOTE (Synthetic Minority Over-sampling Technique), inverse-frequency sample weights and under-sampling the majority classes were tested on the model [14,16].

### 2.7. Model Development

A supervised multiclass classification model based on gradient-boosted decision trees was trained using the XGBoost architecture. This choice was made as XGBoost works well on tabular, mixed-type datasets and can handle non-linear data [17,18,19].

The NACC dataset was split into training, validation and test subsets using a stratified 80/10/10 split to handle class imbalance and with a fixed random seed (random_state = 42) to support reproducibility. Because the NACC dataset contains repeated visits for some participants, the train/validation/test split was performed at the participant level, not the visit level, to avoid leakage of repeated records from the same individual across training and test sets. L1 and L2 regularization was added to reduce overfitting. Extensive hyperparameter tuning was performed using coarse-to-fine grid search.

### 2.8. Statistical Analysis and Model Performance Metrics

Both the loss and accuracy were tracked on the training, validation and test sets to monitor model performance and to adjust hyperparameters. Model performance was monitored using multiple metrics such as accuracy together with class-wise precision, recall, F1-score, macro-F1, weighted-F1, ROC/AUC, balanced accuracy, and confusion matrices. SHAP values were calculated to analyze the most impactful features of the model [20].

A large focus was set on reducing true-positive, false-negative, and minority-class errors because these are clinically important in medical classification tasks [20]. Therefore, class-wise recall and balanced accuracy were emphasized alongside weighted metrics. The SCD class was evaluated separately because it represented the early-stage category but was underrepresented in both the NACC and PSCUH dataset.

Additionally, to track the model’s performance, the prediction confidence levels were tracked together with the predictions. The ECE (Expected Calibration Error) score was used to analyze whether the model becomes overconfident at certain confidence levels and to make sure that the model is well-calibrated [21].

### 2.9. Computational Environment and Software

All analyses were performed in the Google Colab environment (runtime 2025.10) using Python 3.12.13. Data handling and modeling were implemented with NumPy (v2.0.2) and pandas (v2.2.2), while preprocessing, splitting procedures, and performance metrics as well as model interpretability analyses were carried out using scikit-learn (v1.6.1). Model training was conducted with XGBoost (v3.2.0).

### 2.10. Data/Code Availability and Restrictions

NACC data are available only through the official NACC request process and are provided under a Data Use Agreement [22]; therefore, the authors cannot share the dataset. The Latvian retrospective dataset is not publicly available due to ethics committee requirements and patient confidentiality restrictions.

The authors intend to secure the code due to the ongoing patent protection process. Therefore, the full analysis code is not publicly released at the time of the submission. Methodological details, model development, data variables used, preprocessing, processing information as well as missing-value handling rules are reported in the manuscript to support transparency and reproducibility.

### 2.11. The Use of Generative Artificial Intelligence

This manuscript was translated from the original Latvian research materials into English with the assistance of ChatGPT (model: GPT-5.2 Thinking) for translation and language editing. Generative AI was not used to assist in study design, data collection, analysis, or interpretation. The research content, methods, results and conclusions are based on the original study materials and the author’s work.

## 3. Results

### 3.1. Four-Class Classifications on the NACC Dataset: SCD Classification Limitations

The initial model was trained to classify four diagnostic categories in the NACC dataset: normal cognition; subjective cognitive decline (SCD, recorded in NACC data as impaired-not-MCI); MCI; and dementia. Because the classes were highly imbalanced (SCD accounted for only ~4% of records), inverse-frequency class weighting was applied during training to counteract the imbalance. As will be addressed below, the focus was to optimize both overall accuracy and the accuracy of the SCD class, especially as it was the most difficult to classify.

**Figure 1:**
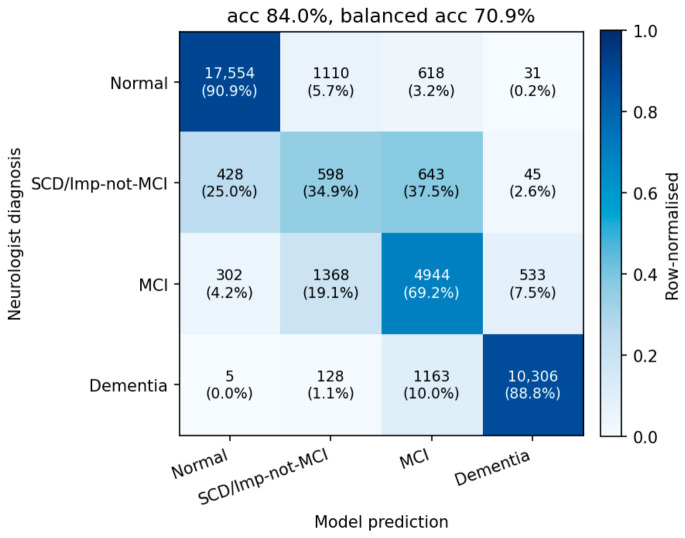
Confusion matrix of the 4-class model (NACC test set).

The weighted four-class model achieved an overall accuracy of 84.0% and a balanced accuracy of 70.9% (macro one-vs-rest AUROC: 0.93). Despite the previously mentioned imbalances, the SCD class remained poorly classified (recall: 34.9%, precision: 18.7%, F1: 0.289), as shown in Figure 1.

To demonstrate that the weighting strategy was not an artifact, the same model was trained with and without class weights (Table 2). Without weighting, the model achieved slightly higher overall accuracy (87.9%); however, it effectively ignored SCD (recall—3.2%, F1—0.06). With weighting, SCD recall improved to 34.9%, but the error simply shifted from recall to precision (F1—0.24). In both settings, normal cognition and dementia were classified well, whereas SCD and, to a lesser degree, MCI were not. Even when taking a subset of the dataset that contained equal amounts of samples of each of the four classes, SCD remained significantly more difficult to classify with only 51.9% accuracy. SCD was therefore found to be intrinsically difficult to separate from the available clinical variables, independent of class imbalance, and is suggested to be very difficult to distinguish by looking at clinical data alone [23].

### 3.2. Initial SHAP Analysis and the Removal of Diagnostic-Assessment-Derived Features

**Figure 2:**
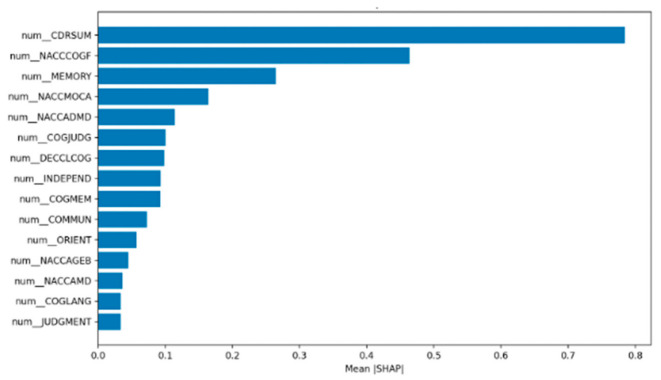
Initial SHAP analysis for the XGBoost model on the NACC test set. CDRSUM (standard CDR sum); NACCCOGF (predominant changes in cognition); MEMORY (assessment of memory); NACCMOCA (MoCA total score); NACCADMD (reported current use of FDA-approved medication for Alzheimer’s disease); COGJUDG (comparison of prior judgment functioning with current judgment status); DECCLCOG (current impairment in cognition); INDEPEND (level of independence); COGMEM (comparison of prior memory functioning with current memory status); ORIENT (orientation); COMMUN (community affairs); NACCAGEB (subject’s age at initial visit); JUDGMENT (judgment and problem-solving); COGLANG (comparison of prior language functioning with current language status); NACCAMD (total number of medications reported at each visit).

The model was trained on 55 features derived from the original dataset. Performing a SHAP analysis on the NACC test set shows that CDRSUM (Standard CDR sum), NACCOGF (predominant changes in cognition) and MEMORY (Uniform Data Set to assess memory) are the leading features for diagnosis (Figure 2). These features are strong indicators because they can capture the main memory-related cognition changes and the degree and severity of how it affects a person’s everyday life [24].

Figure 2 shows the global SHAP feature importance profile of the XGBoost model on the NACC test set, ranked by mean absolute SHAP value (Mean |SHAP|). CDRSUM was the most influential feature, with a mean |SHAP| of 0.8, followed by NACCCOGF at 0.46 and MEMORY at 0.25. A second tier of features included NACCMOCA—0.15 and NACCADMD—0.11. The remaining variables each had mean |SHAP| values below 0.10. These findings indicate that the model predictions were mainly calculated by using a small number of cognitive and functional measures.

**Figure 3:**
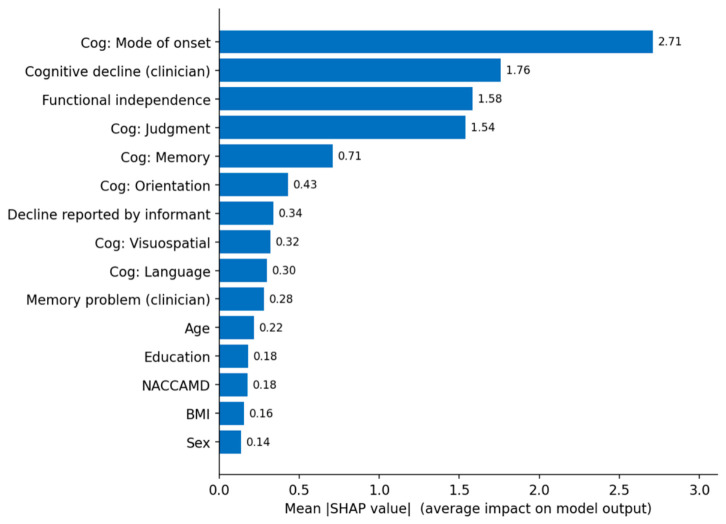
Retrained model’s SHAP analysis on the NACC test set.

However, the features themselves are closely related variables used in routine clinical diagnostic assessment. To quantify the model’s dependence on this information, all diagnostic-assessment-derived features were removed (Table 3), and the four-class model was retrained. Another SHAP analysis was performed to see the leading variables, seen in Figure 3.

Figure 3 shows that the mode of onset of cognitive symptoms—gradual, subacute or abrupt—was the most influential feature, with a mean |SHAP| value of 2.71, followed by the clinician’s subjective assessment of the patient’s impairment in cognition at 1.76, the level of independence at 1.58 and comparison of prior judgment functioning with current judgment status at 1.54. These findings indicate that, even after the formal cognitive test scores and CDR variables were removed, model predictions remained dominated by the clinician’s structured cognitive-symptom ratings (mode of onset of cognitive symptoms; clinician’s subjective opinion of patient experiencing meaningful memory, judgment, orientation, visuospatial and/or language decline) together with functional independence. Meanwhile, the variables sex, BMI, age and NACCAMD (the total number of medications used) were less impactful as analyzed in the SHAP analysis.

**Figure 4:**
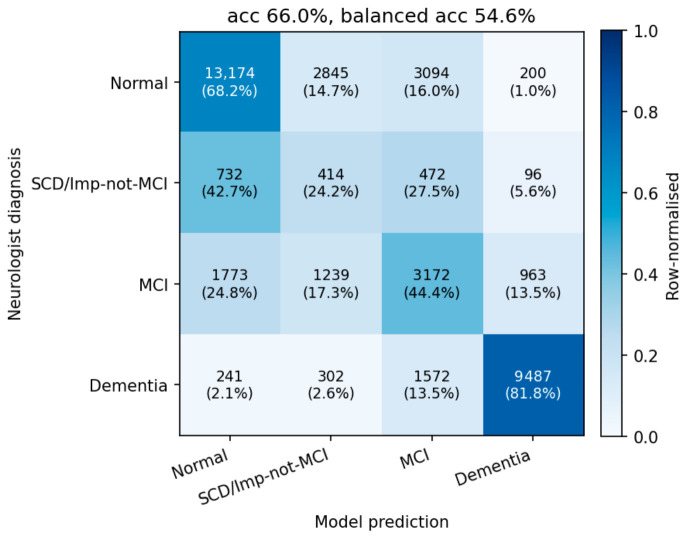
Confusion matrix of the 4-class model after removing the diagnostic-assessment-derived features (NACC test set).

When adjusting the model to train without any diagnostic-assessment-derived features, accuracy dropped significantly across all classes, as can be seen in Figure 4. The model struggles again with the SCD class as before, but also the MCI class is misclassified way more than before. Since the diagnostic-assessment-derived features were routinely used clinical rules or scores, for example, MoCA and/or CDR domains, the model now needs to predict the diagnosis using independent rules that lead to lower accuracy (66%) and balanced accuracy (54.6%).

### 3.3. Three-Class Classification with and Without the Diagnostic-Assessment-Derived Features

**Figure 5:**
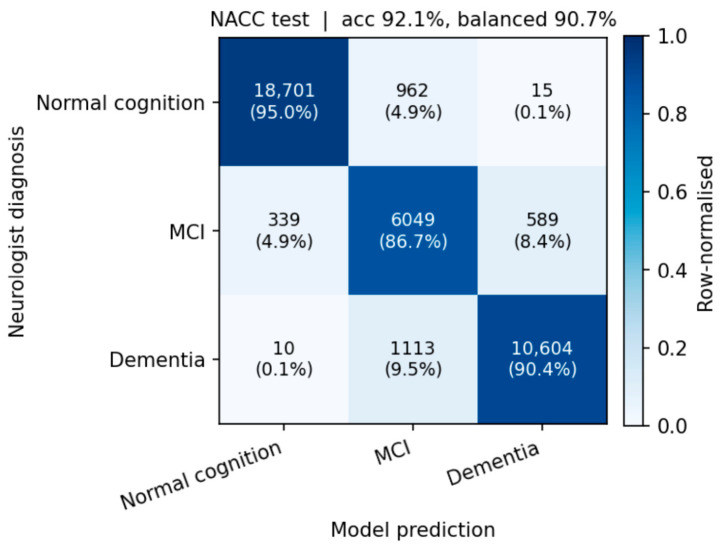
Confusion matrix of the three-class classification model with diagnostic-assessment-derived features (NACC test set).

Because SCD could not be reliably identified in the four-class setting, the primary analysis was reframed as a three-class classification (normal cognition, MCI, and dementia). In the full feature set model, the classifier reproduced close agreement with the neurologist-assigned diagnosis. Overall, the accuracy was 91.5%, and balanced accuracy was 90.2% (Table 4; Figure 5).

**Figure 6:**
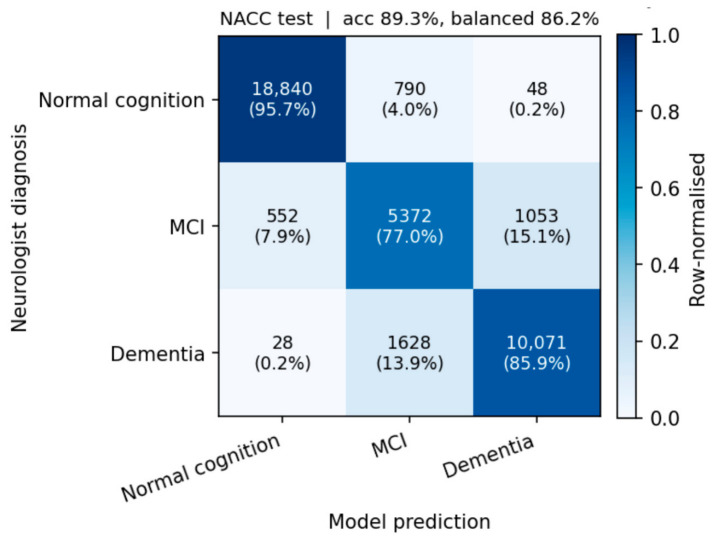
Confusion matrix of the three-class classification model without diagnostic-assessment-derived variables.

When the clinician-judgment-derived features were removed, the overall accuracy fell to 89.3% and balanced accuracy to 86.2% (Table 4; Figure 6).

These findings indicate that the diagnostic-assessment-derived features substantially improved the classification performance. However, the model retained meaningful, above-chance discriminatory ability after their removal—suggesting that the model was not merely re-encoding the diagnostic rules.

**Figure 7:**
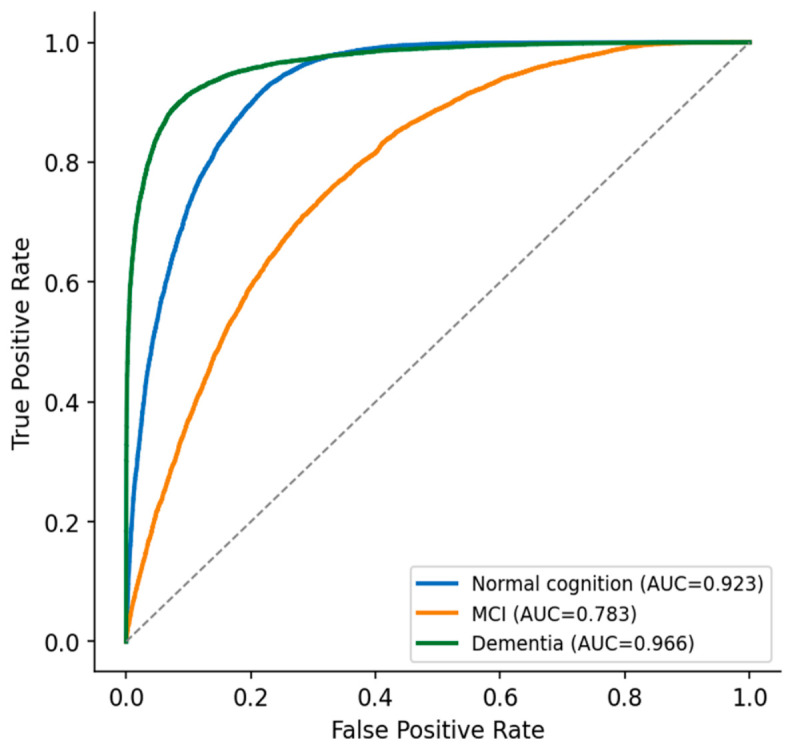
One-vs-rest ROC curves of the three-class classification model without diagnostic-assessment-derived variables (NACC test set).

This type of result can also be seen in Figure 7. That shows the one-vs-rest ROC curves for the three diagnostic classes on the NACC dataset.

All three curves lie above the chance diagonal, but they are well-separated one from another. The dementia curve is the strongest, rising high towards the upper-left corner and achieving an AUC of 0.966, indicating that high true-positive rates are reached with very few false positives. A similar outcome can be seen with normal cognition (AUC—0.923). The MCI curve is visibly lower than the other two, particularly in the mid-range of false-positive rates (AUC—0.783). The macro-average across all three classes is 0.891.

### 3.4. External Validation of the Latvian (PSCUH) Cohort Using the Three-Class Classification Without Diagnostic-Assessment-Derived Features

The three-class classification model (without diagnostic-assessment-derived features) was applied to the external Latvian cohort. The cohort was a dementia-clinic referral population and contained essentially no normal-cognition cases and only two SCD cases. Therefore, the most clinically meaningful external evaluation is the distinction between dementia and MCI.

**Figure 8:**
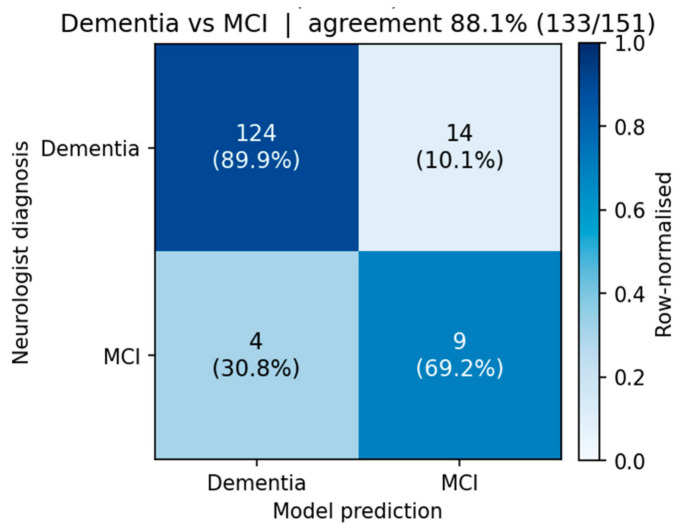
Confusion matrix of the external Latvian cohort without diagnostic-assessment-derived variables.

The model agreed with the neurologist-assigned diagnosis in 88.1% of the cases (133/151), with dementia sensitivity of 89.9% (124/138) and MCI sensitivity of 69.2% (9/13) (Figure 8). These findings indicate that the external agreement was largely preserved for dementia and remained moderate for MCI, even after the removal of diagnostic-assessment-derived features. Additionally, it is very important to note that there were no misclassifications of dementia or MCI as normal cognition. Based on this fact, in Figure 8, normal cognition was not included in the confusion matrix.

## 4. Discussion

The aim of the study was to develop and externally evaluate an ML-based classification model for cognitive status categories using structured available clinical and cognitive-assessment data. The research does not demonstrate a direct comparison between the ML model and a physician but rather evaluates how well a supervised model trained on the NACC dataset agrees with clinician-assigned diagnoses. This distinction is important because the physician’s diagnosis acted as the reference standard and not as a comparator.

One of the most important findings was the model’s overall high diagnostic accuracy, despite the limited clinical information. However, its ability to distinguish between two clinically similar and adjacent diagnoses, such as subjective cognitive decline and mild cognitive impairment, was lower since SCD is a clinically broad category and may reflect other psychiatric symptoms, depression, sleep disturbance or vascular factors rather than early dementia complaints. By being the minority class in the dataset, it would indicate that a larger dataset group needs to be implemented to increase the accuracy of the model when overlapping conditions must be differentiated.

The most appropriate role of ML at this current stage of technological development may be as a supportive tool for the physician rather than an independent diagnostic instrument. Based on the present findings, the model has the potential to be a structured decision supporter to organize routinely collected cognitive and clinical variables, assisting clinicians when diagnosing a patient as well as supporting future development of standardized clinical decision support systems.

When comparing the assessments of both parties, an interesting pattern was observed. The algorithm more often classified patients into more severe cognitive categories, suggesting a cautious strategy aimed at reducing the risk of incorrectly classifying findings as normal. In contrast, the neurologist’s reasoning was based more on the observation of symptom dynamics over time and the possible progression of the diagnosis. This often delayed the recognition of the diagnosis; therefore, the patients transitioned from early cognitive complaints to more pronounced symptoms, such as memory loss and social functioning difficulties.

Several limitations should also be acknowledged. The number of patients and large dataset class-imbalance between diagnostic categories, particularly SCD and MCI, likely contributed to the model’s limited ability to distinguish seemingly adjacent diagnoses. With only two SCD cases available in the PSCUH dataset, a meaningful evaluation of the model’s SCD categorization is impossible. The retrospective nature of the research provides a useful overview of how diagnoses are established in practice; however, prospective, larger and more balanced studies could provide more targeted questions and obtain specific information to improve the ML model’s accuracy, for example, evaluating longitudinal SCD-to-MCI conversion to identify higher risk groups, as well as to reduce the proportion of missing values. Also, because cognitive testing such as the CDR, MMSE, and MoCA is part of routine clinical assessment, the first model partly reproduced existing diagnostic rules rather than identifying a new and independent pattern. Another limitation is the “black box” limitation as it is difficult for the researchers to fully interpret and represent the model’s internal reasoning strategy and decision-making logic in a completely transparent way.

## 5. Conclusions

This research developed an XGBoost-based ML classification model using the NACC dataset, excluding the utilization of biomarkers or neuroimaging, and externally evaluated it in a Latvian retrospective cohort. The results suggest that the aims and objectives of this research were accomplished. The ML model demonstrated the potential clinical embracement of such technology as a supportive tool, rather than a standalone diagnostic system, in cognitive assessment and diagnostic decision-making, especially when used in combination with physicians. The SHAP analysis indicated that the primary driving factors for successful diagnostic predictions were driven by cognitive test results and other clinically relevant factors, supporting the model’s capability of interpreting clinical data.

However, the research also showed that the model’s performance decreased when trying to distinguish clinically similar and adjacent diagnoses, highlighting the importance of continuous physician assessment of ML-based predictions.

Overall, the ML model seems to be a promising contributing tool for cognitive assessment in clinical settings, but further prospective research needs to confirm its safety, efficiency and practical value in real-time clinical practice.

## 6. Patents

The authors are pursuing a patent protection at the time of the manuscript submission.

## Figures and Tables

**Table 1:** Baseline characteristics of the NACC model-development dataset and the external PSCUH dataset.

Characteristic	NACC Total	NACC Training Set	NACC Validation Set	NACC Test Set	PSCUH
Participants, *n*	54025	43220	5402	5403	153
Age at visit, mean	74.8	74.8	74.8	74.7	80.3
Female sex, %	58.1	58.2	57.2	58.8	68.6
Male sex, %	41.9	41.8	42.8	41.2	31.4
Education years, mean	15.6	15.6	15.6	15.6	13.6
Normal cognition, %	48.9	48.9	48.5	49.1	0
SCD, %	4.4	4.4	4.4	4.3	1.3
MCI, %	17.5	17.4	17.9	17.9	8.5
Dementia, %	29.2	29.2	29.2	28.8	90.2
MoCA, mean	23.5	23.5	23.3	23.6	20.4
MoCA missing, %	65.4	65.5	64.5	65.8	37.3
MMSE, mean	25.5	25.5	25.6	25.6	-
MMSE missing, %	50.6	50.5	51.6	50.5	100
CDR sum, mean	2.7	2.7	2.7	2.7	2.8
CDR sum missing, %	0	0	0	0	0
Hypertension, %	54.9	54.9	55.5	54.7	88.2
Diabetes mellitus, %	13.9	14.0	13.8	13.4	20.9
Hypercholesterolemia, %	56.1	56.0	56.5	56.3	44.4
Stroke history, %	5.6	5.7	5.4	5.2	18.3
Depression in last 2 years, %	29.7	29.6	30.3	30.2	7.8

**Table 2:** Effect of class weighting on SCD detection (4-class model, NACC dataset).

4-Class Model	Model Accuracy, %	SCD F1 Score
Unweighted (baseline)	87.9	0.06
Inverse-frequency weighted	84.2	0.24
Balanced class dataset	72.3	0.51

**Table 3:** Feature groups included in the initial model and retrained model.

Feature Group	Variables	Initial Model	Retrained Model
Sociodemographic	Age, sex, education, marital status, living status, economic situation	Included	Included
General medical history	Hypertension, diabetes mellitus, stroke history, heart attack/cardiac arrest history, hypercholesterolemia, other comorbidities	Included	Included
Psychiatric	Depression and anxiety	Included	Included
Patient- or informant-reported cognitive complaints	Subjective cognitive, memory complaints	Included	Included
General medication, excluding Alzheimer’s disease-specific medication	Total number of medications	Included	Included
Formal cognitive tests	MoCA, MMSE scores	Included	Excluded
CDR	Sum of CDR, CDR orientation/memory/judgment/community affairs/hobbies/personal care	Included	Excluded
Clinician structured assessment of cognitive impairment	Assessment of memory, judgment, orientation, language, predominant changes in memory	Included	Excluded

**Table 4:** Three-class classification model performance with and without the diagnostic-assessment-derived variables (NACC test set).

Class	Feature Set	Sensitivity, %	Specificity, %	Positive Predicted Value, %	F-1 Score	AUROC
Normal cognition	With	94.5	98.1	98.1	0.96	0.993
Normal cognition	Without	78.0	87.7	87.0	0.82	0.923
MCI	With	86.3	92.8	72.7	0.72	0.961
MCI	Without	57.3	81.2	40.4	0.47	0.783
Dementia	With	89.6	97.7	94.5	0.92	0.991
Dementia	Without	82.6	95.6	89.2	0.86	0.966

## Data Availability

Restrictions apply to the availability of these data. Data was obtained from the National Alzheimer’s Coordinating Center (NACC) and are available at (https://www.naccdata.org/) with the permission of the NACC. The Latvian retrospective dataset is not publicly available due to ethics committee requirements and patient confidentiality restrictions.

## Abbreviations

The following abbreviations are used in this manuscript:

AI	Artificial intelligence
AD	Alzheimer’s disease
ADRCs	Alzheimer’s Disease Research Centers
CDR	Clinical dementia rating
ECR	Expected Calibration Error
ICD	International Classification of Diseases
MCI	Mild cognitive impairment
MMSE	Mini-mental state examination
ML	Machine learning
MoCA	Montreal cognitive assessment
NACC	National Alzheimer’s Coordinating Center
NIA	National Institute of Aging
NaN	Not a number
PSCUH	Pauls Stradins Clinical University Hospital
QR	Quick response
SCD	Subjective cognitive decline
SHAP	Shapley Additive Explanations
SMOTE	Synthetic Minority Over-sampling Technique
WHO	The World Health Organization
